# Opening of Medical Schools

**Published:** 1859-11

**Authors:** 


*Opening of Medical Schools.*—The institutions for medical instruction have now fairly commenced all over the country; and, if we may judge from those in the Metropolis, and what few others we have heard from, with better prospects than for many years. The number of young men about entering the medical profession is undoubtedly on the increase. The New York colleges are all fuller than at the same time last year; there are more students than usual in Philadelphia; and one or two of the Southern schools which we have heard from, are also beginning with larger classes than usual. Now is the time, when students are becoming numerous, to guard the profession from the admission of unqualified and unsuitable men; and this we must do by a rigid examination into the qualifications of every candidate for a degree, increasing, at the same time, our zeal in teaching all departments, both elementary and practical. Let every school make the most of its advantages. It will then be the student's fault if he be not qualified to practice his profession after having pursued the required course of study, and culpable negligence or willful wrong on the part of any faculty which confers a degree upon an unqualified person. We have heard indirectly from the Buffalo school, in which, though now connected with another institution, we will always take a deep interest, and learn that its prospects for the coming session are good.

